# Metagenomic assembled dataset of potentially polyethylene terephthalate-degrading microcosms enriched from seawater, cow dung, and landfill soil

**DOI:** 10.1016/j.dib.2025.111671

**Published:** 2025-05-15

**Authors:** Aubrey Dickon Chigwada, Henry Joseph Oduor Ogola, Memory Tekere

**Affiliations:** Department of Environmental Science, College of Agricultural and Environmental Sciences, University of South Africa, Roodepoort, Gauteng, South Africa

**Keywords:** Metagenome-assembled genomes, Polyethylene terephthalate (PET), PET degradation, Biodegradation, Pollution management

## Abstract

We present a dataset of 99 prokaryotic metagenome-assembled genomes (MAGs) derived from 180-day culture-enrichment microcosms of seawater, landfill soil, and cow dung, with polyethylene terephthalate (PET) as the sole carbon source. The recovered MAGs met the medium-to-high quality standards of the Minimum Information for Metagenome-Assembled Genomes (MIMAG) criteria with completeness ranging from 76.5% to 100% and low contamination levels (<10%). The majority of the MAGs were obtained from seawater (52), followed by cow dung (28), and landfill soil (19). Additionally, the dataset includes detailed DRAM (Distilled and Refined Annotation of Metabolism) functional profiles of the MAGs, which highlight the potential role of these microorganisms in the biodegradation of PET polymers. This genomic data provides valuable reference information on bacteria and archaea with the potential capacity to biodegrade plastic, contributing to our understanding of microbial plastic biodegradation.

## 1 Value of the Data

• The MAGs presented here present a significant contribution to reference genomes within databases focused on PET degradation, specifically from diverse environments such as seawater, cow dung, and landfill soil.
• These datasets provide a valuable resource for future comparative analyses of gene sets in microorganisms harboring PET bioremediation genes, facilitating insights into functional capabilities and metabolic pathways associated with plastic degradation.
• When compared to MAGs from similar environmental settings, these genomes offer valuable information regarding evolutionary adaptations and microbial responses to PET contamination.
• Many of the recovered MAGs align with well-characterized species, making them suitable for inclusion in pan-genomic analyses, which can enhance our understanding of genetic diversity within PET-degrading microbial communities.
• The MAGs exhibit a high degree of novelty in both taxonomic classification and functional annotation, highlighting previously unrecognized microbial taxa and metabolic pathways with potential roles in plastic biodegradation.

## 2 Background

Microbial biodegradation of PET presents a promising approach for managing plastic pollution in the environment. In this context, this study aimed to enrich microbial consortia from landfill soil, seawater, and cow dung with the capacity to biodegrade PET. Despite the growing interest in PET-degrading microorganisms, there is a limited body of published molecular genetic data on the bacteria and archaea involved in this process, particularly from such diverse environments. To address this gap, we employed MAG techniques to generate genomic data that would enable the identification and functional annotation of microbial genes associated with PET biodegradation. This approach provides valuable insights into the genetic and functional properties of PET-degrading microbes, which are critical for advancing bioremediation strategies. Table 1 represents the specifications of the data.

**Table 1:** Specifications of the Data.

Subject	Microbiology: Biotechnology Remediation
Specific subject area	Plastic-degrading microbial communities
Type of data	FASTA files. tables. and figures
Data collection	Composite samples were collected from landfill soil, seawater, and cow dung. Samples were used as inocula in 180-day lab enrichment experiments using PET as the sole carbon source. Post-enrichment, DNA was extracted and sequenced using the DNBSEQ-G400. Reads were assembled with metaSPAdes and contigs binned with MaxBin, metaBAT2, and CONCOCT software. Recovered MAGs were taxonomically classified and annotated using GTDB-tk and DRAM.
Data source location	**Landfill soil samples** City/Town/Region: Mogale City, Gauteng Country: South Africa Latitude and longitude: (26.00 S 27.66 E) **Seawater samples** City/Town/Region: Durban, KwaZulu Natal Country: South Africa Latitude and longitude: (29.83 S 31.03 E) **Cow dung samples** City/Town/Region: Pretoria, Gauteng Country: South Africa Latitude and longitude: (26.10 S 27.02 E)
Data accessibility	Repository name: NCBI Genbank Sequence Read Archive (SRA) Data identification number: NCBI Bioproject ID PRJNA1081682 Direct URL to data: https://www.ncbi.nlm.nih.gov/bioproject/PRJNA1081682 [1]

## 3 Data Description

**Fig. 1:**
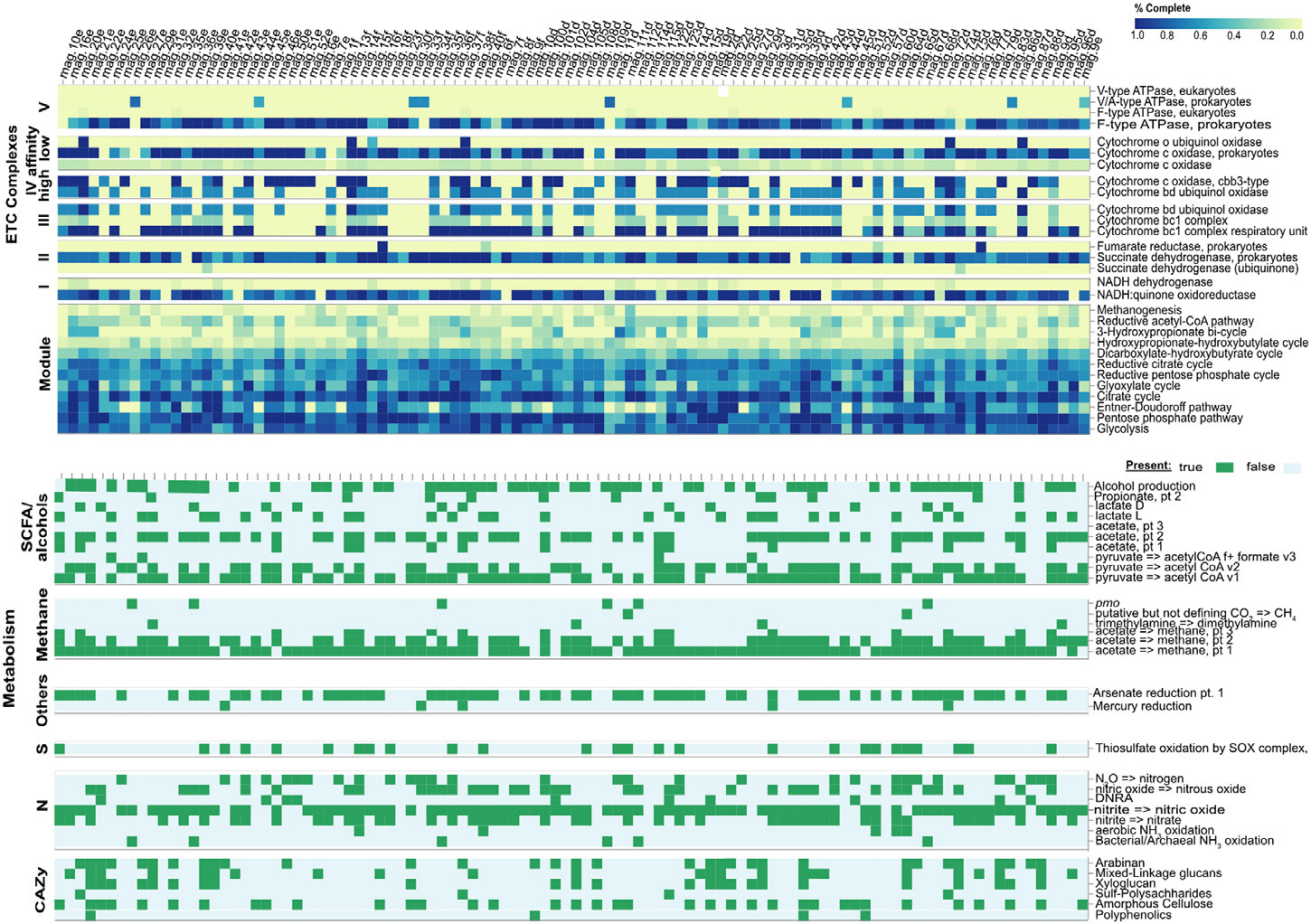
Heatmap showing the DRAM functional annotation profiles of 99 MAGs recovered after 180 days of enrichment using polyethylene terephthalate (PET) as the sole carbon source in microcosms derived from seawater (f), cow dung (e), and landfill soil (d).

This dataset comprises MAGs recovered from microbial communities originating from seawater, landfill soil, and cow dung. These communities were enriched under laboratory conditions for 180 days using PET as the sole carbon source in microcosms. A comprehensive summary of the recovered MAGs is presented in Table 2, with genome completeness ranging from 76.5% to 100% and contamination levels remaining below 10%, in accordance with MIMAG standards [2]. Functional gene annotations for these MAGs are visualized as a heatmap in Fig. 1.

**Table 2:** A list of 99 MAGs is available in the dataset sourced from 180-day culture-enrichment microcosm of seawater, cow dung, and landfill soil using PET as the sole carbon source. GTDB-Tk was used to generate the taxonomic classification. Functional novelty is reflected by the percentage of predicted genes.

MAG	Taxonomy	Comp† (%)	Cont∞ (%)	Genome size (bp)	Contigs	N50 (bp)	GC (%)	ˁCDS	NCBI Genbank Assembly	GTDB-Tk Taxonomy
**MAGs from Landfill soil microcosms**										
mag.1d	*Flavobacteriales bacterium*	100	0	3376098	24	253259	60.7	2814	GCA_044508845.1	k_Bacteria;p_Bacteroidetes;c_Flavobacteriia;o_Flavobacteriales
mag.36d	*Rubrivivax sp.*	99.53	1.64	4795103	67	111151	71.6	4354	GCA_044508835.1	k_Bacteria;p_Proteobacteria;c_Betaproteobacteria; o_Burkholderiales;f_Rubrivivax
mag.37d	*Sphingomonadales bacterium*	99.3	0.93	3681160	67	94594	64.2	3593	GCA_044508875.1	k_Bacteria;p_Proteobacteria;c_Alphaproteobacteria; o_Sphingomonadales
mag.8d	*Propionibacteriaceae bacterium*	99.22	0.69	3250709	48	119547	69.9	3018	GCA_044508675.1	k_Bacteria;p_Actinobacteria;c_Actinobacteria; o_Actinomycetales;f_Propionibacteriaceae
mag.14d	*Burkholderiales bacterium*	98.29	2.19	3995508	82	126785	69.3	3799	GCA_044508645.1	k_Bacteria;p_Proteobacteria;c_Betaproteobacteria; o_Burkholderiales
mag.35d	*Nitrosomonas sp.*	97.63	0.12	2644038	84	49300	49.7	2462	GCA_044508755.1	k_Bacteria;p_Proteobacteria;c_Betaproteobacteria; o_Nitrosomonadales;f_Nitrosomonadaceae;g_Nitrosomonas
mag.23d	*Planctomycetaceae bacterium*	97.16	0.57	3262784	8	975063	64.1	2731	GCA_044508635.1	k_Bacteria;p_Planctomycetes;c_Planctomycetia; o_Planctomycetales;f_Planctomycetaceae
mag.34d	*Alcaligenaceae bacterium*	96.89	1.72	3964565	106	59435	68.4	3649	GCA_044508625.1	k_Bacteria;p_Proteobacteria;c_Betaproteobacteria; o_Burkholderiales;f_Alcaligenaceae
mag.13d	*Comamonadaceae bacterium*	95.77	0.56	4842164	281	26881	71.1	4762	GCA_044508595.1	k_Bacteria;p_Proteobacteria;c_Betaproteobacteria; o_Burkholderiales;f_Comamonadaceae
mag.9d	*Lysobacter sp.*	95.47	0.78	2389426	51	114988	70.4	2247	GCA_044508555.1	k_Bacteria;p_Proteobacteria;c_Gammaproteobacteria; o_Xanthomonadales;f_Xanthomonadaceae;g_Lysobacter
mag.18d	*Chloroflexota bacterium*	94.81	0.94	3616338	414	11819	62	3371	GCA_044508575.1	k_Bacteria;p_Chloroflexi
mag.40d	*Hyphomicrobiales bacterium*	94.75	2.82	3550720	326	16436	66.4	3597	GCA_044508525.1	k_Bacteria;p_Proteobacteria;c_Alphaproteobacteria;o_Rhizobiales
mag.15d	*Bradyrhizobiaceae bacterium*	92.52	6.09	3771253	541	9871	62.7	3974	GCA_044508545.1	k_Bacteria;p_Proteobacteria;c_Alphaproteobacteria; o_Rhizobiales;f_Bradyrhizobiaceae
mag.7d	*Clavibacter sp.*	91.02	1.91	2242882	190	19407	69.7	2343	GCA_044508495.1	k_Bacteria;p_Actinobacteria;c_Actinobacteria; o_Actinomycetales;f_Microbacteriaceae;g_Clavibacter
mag.30d	*Deinococcaceae bacterium*	90.68	1.27	3022882	58	105559	73.2	2680	GCA_044508445.1	k_Bacteria;p_Deinococcus-Thermus;c_Deinococci; o_Deinococcales;f_Deinococcaceae
mag.16d	*Mycolicibacterium sp.*	90.55	3.16	6732645	313	34488	67.3	6691	GCA_044508435.1	k_Bacteria;p_Actinobacteria;c_Actinobacteria; o_Actinomycetales;f_Mycobacteriaceae;g_Mycobacterium
mag.38d	*Mycolicibacterium sp.*	87.41	1.32	7422144	455	28336	65.7	7619	GCA_044508465.1	k_Bacteria;p_Actinobacteria;c_Actinobacteria; o_Actinomycetales;f_Mycobacteriaceae;g_Mycobacterium
mag.33d	*Deinococcaceae bacterium*	86.33	2.97	2987734	122	49664	69.6	2707	GCA_044508425.1	k_Bacteria;p_Deinococcus-Thermus;c_Deinococci; o_Deinococcales;f_Deinococcaceae
mag.6d	*Mycobacterium sp.*	85.34	1.79	6320813	295	39843	65.4	6503	GCA_044508395.1	k_Bacteria;p_Actinobacteria;c_Actinobacteria; o_Actinomycetales;f_Mycobacteriaceae;g_Mycobacterium
**MAGs from Cow dung microcosms**										
mag.26e	*Nitrosopumilales archaeon*	99.03	1.94	2271978	109	35412	48.7	2686	GCA_044508385.1	k_Archaea;p_Thaumarchaeota;c_Nitrosopumilales; o_Nitrosopumilales
mag.42e	*Propionibacteriaceae bacterium*	98.7	0.87	3608623	96	80213	69.8	3457	GCA_044508365.1	k_Bacteria;p_Actinobacteria;c_Actinobacteria; o_Actinomycetales;f_Propionibacteriaceae
mag.6e	*Microbacterium sp.*	97.98	1.77	3302734	44	154039	70.8	3169	GCA_044508325.1	k_Bacteria;p_Actinobacteria;c_Actinobacteria; o_Actinomycetales;f_Microbacteriaceae;g_Microbacterium
mag.21e	*Actinomadura sp*.	97.86	2.14	7363213	240	47228	72.9	6798	GCA_044508335.1	k_Bacteria;p_Actinobacteria;c_Actinobacteria; o_Actinomycetales;f_Thermomonosporaceae;g_Actinomadura
mag.44e	*Hyphomicrobiaceae bacterium*	97.59	7.22	3896203	233	33612	64.6	3814	GCA_044508295.1	k_Bacteria;p_Proteobacteria;c_Alphaproteobacteria; o_Rhizobiales;f_Hyphomicrobiaceae
mag.20e	*Sphingomonadales bacterium*	96.93	4.4	3727519	354	15709	64.4	3926	GCA_044508285.1	k_Bacteria;p_Proteobacteria;c_Alphaproteobacteria; o_Sphingomonadales
mag.51e	*Acidobacteriota bacterium*	96.11	6.48	4456932	271	29452	70.1	3992	GCA_044508265.1	k_Bacteria;p_Acidobacteria
mag.52e	*Flavobacteriales bacterium*	95.41	1.08	3189974	280	15746	60.8	2911	GCA_044508195.1	k_Bacteria;p_Bacteroidetes;c_Flavobacteriia; o_Flavobacteriales
mag.35e	*Actinomycetota bacterium*	94.59	2.74	3263226	237	20509	71.7	3189	GCA_044508215.1	k_Bacteria;p_Actinobacteria;c_Actinobacteria
mag.45e	*Nitrosomonas sp.*	94.58	0.48	2154330	117	27902	49.7	1937	GCA_044508205.1	k_Bacteria;p_Proteobacteria;c_Betaproteobacteria; o_Nitrosomonadales;f_Nitrosomonadaceae;g_Nitrosomonas
mag.50e	*Planctomycetaceae bacterium*	94.32	0	4187701	351	20621	71.5	3583	GCA_044508185.1	k_Bacteria;p_Planctomycetes;c_Planctomycetia; o_Planctomycetales;f_Planctomycetaceae
mag.36e	*Nitrosomonas sp.*	94.08	0.63	2605714	130	29778	49.7	2447	GCA_044508165.1	k_Bacteria;p_Proteobacteria;c_Betaproteobacteria; o_Nitrosomonadales;f_Nitrosomonadaceae;g_Nitrosomonas
mag.24e	*Actinomycetales bacterium*	93.93	1.16	2022824	36	98372	63.8	1963	GCA_044508135.1	k_Bacteria;p_Actinobacteria;c_Actinobacteria; o_Actinomycetales
mag.29e	*bacterium*	93.06	0.93	2289810	1	2289810	55.2	2249	GCA_044508115.1	k_Bacteria
mag.46e	*Burkholderiales bacterium*	92.93	3.4	3635072	219	28456	70.2	3457	GCA_044508095.1	k_Bacteria;p_Proteobacteria;c_Betaproteobacteria; o_Burkholderiales
mag.31e	*Acidobacteriota bacterium*	92.31	4.46	3740637	159	41872	68.5	3396	GCA_044508085.1	k_Bacteria;p_Acidobacteria
mag.39e	*Lysobacterales bacterium*	91.9	3.58	4495370	256	38443	61.7	4029	GCA_044508065.1	k_Bacteria;p_Proteobacteria;c_Gammaproteobacteria; o_Xanthomonadales
mag.25e	*Myxococcales bacterium*	91.75	4.11	6308533	563	17695	67.5	5626	GCA_044508025.1	k_Bacteria;p_Proteobacteria;c_Deltaproteobacteria; o_Myxococcales
mag.32e	*Sphingomonadales bacterium*	91.41	6.28	3201100	245	23568	68.9	3260	GCA_044508015.1	k_Bacteria;p_Proteobacteria;c_Alphaproteobacteria; o_Sphingomonadales
mag.43e	*Rubrivivax sp.*	91.03	4.77	4538033	452	17172	71.8	4522	GCA_044508005.1	k_Bacteria;p_Proteobacteria;c_Betaproteobacteria; o_Burkholderiales;f_Rubrivivax
mag.27e	*Mycolicibacterium sp.*	89.13	2.61	6656724	489	20841	67.3	6705	GCA_044507965.1	k_Bacteria;p_Actinobacteria;c_Actinobacteria; o_Actinomycetales;f_Mycobacteriaceae;g_Mycobacterium
mag.9e	*Deinococcaceae bacterium*	88.66	2.73	2658142	262	15448	71.6	2635	GCA_044507975.1	k_Bacteria;p_Deinococcus-Thermus;c_Deinococci; o_Deinococcales;f_Deinococcaceae
mag.16e	*Hyphomicrobiales bacterium*	88.36	2.32	4197916	341	18631	68.7	4163	GCA_044507925.1	k_Bacteria;p_Proteobacteria;c_Alphaproteobacteria; o_Rhizobiales
mag.7e	*Nitrospiraceae bacterium*	87.34	4.12	4279919	696	7910	57.9	4592	GCA_044507885.1	k_Bacteria;p_Nitrospirae;c_Nitrospira; o_Nitrospirales;f_Nitrospiraceae
mag.40e	*Hyphomicrobiales bacterium*	87	0.55	2739656	178	27924	64.9	2860	GCA_044507875.1	k_Bacteria;p_Proteobacteria;c_Alphaproteobacteria; o_Rhizobiales
mag.10e	*Rubrivivax sp.*	86.64	2.93	3578849	665	6943	69.9	3908	GCA_044507895.1	k_Bacteria;p_Proteobacteria;c_Betaproteobacteria; o_Burkholderiales;f_Rubrivivax
mag.22e	*Opitutales bacterium*	79.52	3.19	3677598	896	4785	62.9	3863	GCA_044507865.1	k_Bacteria;p_Verrucomicrobia;c_Opitutae;o_Opitutales
mag.41e	*Chloroflexota bacterium*	77.1	8.49	5055509	1471	3860	66.2	5791	GCA_044507845.1	k_Bacteria;p_Chloroflexi
**MAGs from seawater microcosms**										
mag.25f	*Flavobacteriales bacterium*	100	0	3339406	23	253259	60.7	2794	GCA_044507815.1	k_Bacteria;p_Bacteroidetes;c_Flavobacteriia; o_Flavobacteriales
mag.88f	*Flammeovirgaceae bacterium*	99.7	0.89	5342577	58	158042	50.4	4403	GCA_044507775.1	k_Bacteria;p_Bacteroidetes;c_Cytophagia; ;o_Cytophagales;f_Flammeovirgaceae
mag.72f	*Sphingomonadales bacterium*	99.64	2.45	3698613	76	101760	64.2	3644	GCA_044507765.1	k_Bacteria;p_Proteobacteria;c_Alphaproteobacteria; o_Sphingomonadales
mag.38f	*Actinomadura sp.*	99.24	2.67	7333541	251	46853	72.9	6773	GCA_044507795.1	k_Bacteria;p_Actinobacteria;c_Actinobacteria; o_Actinomycetales;f_Thermomonosporaceae;g_Actinomadura
mag.109f	*Nitrosopumilales archaeon*	99.03	0.97	2325507	55	69305	48.6	2720	GCA_044507735.1	k_Archaea;p_Thaumarchaeota;c_Nitrosopumilales; o_Nitrosopumilales
mag.100f	*Alcaligenaceae bacterium*	98.84	2.29	3937094	115	58276	68.4	3633	GCA_044507725.1	k_Bacteria;p_Proteobacteria;c_Betaproteobacteria; o_Burkholderiales;f_Alcaligenaceae
mag.122f	*Ignavibacteriales bacterium*	98.6	0	3711771	31	225332	35.4	3176	GCA_044507695.1	k_Bacteria;p_Ignavibacteriae;c_Ignavibacteria; o_Ignavibacteriales
mag.111f	*Hyphomicrobiaceae bacterium*	97.98	1.25	3725183	43	168336	64.6	3481	GCA_044507625.1	k_Bacteria;p_Proteobacteria;c_Alphaproteobacteria; o_Rhizobiales;f_Hyphomicrobiaceae
mag.19f	*Opitutales bacterium*	97.97	3.38	4305176	112	82772	62.8	3749	GCA_044507675.1	k_Bacteria;p_Verrucomicrobia;c_Opitutae;o_Opitutales
mag.60f	*Burkholderiales bacterium*	97.83	2.4	4000918	121	85414	69.2	3826	GCA_044507665.1	k_Bacteria;p_Proteobacteria;c_Betaproteobacteria; o_Burkholderiales
mag.123f	*Rubrivivax sp.*	97.43	1.64	4621223	112	68115	71.7	4241	GCA_044507635.1	k_Bacteria;p_Proteobacteria;c_Betaproteobacteria; o_Burkholderiales;f_Rubrivivax
mag.104f	*Actinomycetales bacterium*	97.22	2.22	6443066	165	66667	71.8	5778	GCA_044507605.1	k_Bacteria;p_Actinobacteria;c_Actinobacteria; o_Actinomycetales
mag.57f	*Planctomycetaceae bacterium*	97.16	1.7	3267673	30	177946	64.1	2746	GCA_044507575.1	k_Bacteria;p_Planctomycetes;c_Planctomycetia; o_Planctomycetales;f_Planctomycetaceae
mag.87f	*Planctomycetaceae bacterium*	96.59	0	4257266	275	28068	71.5	3610	GCA_044507535.1	k_Bacteria;p_Planctomycetes;c_Planctomycetia; o_Planctomycetales;f_Planctomycetaceae
mag.10f	*Actinomycetota bacterium*	96.58	1.71	3224022	192	29718	71.7	3101	GCA_044507565.1	k_Bacteria;p_Actinobacteria;c_Actinobacteria
mag.9f	*Burkholderiales bacterium*	96.33	3.47	3662353	231	25781	70.2	3483	GCA_044507525.1	k_Bacteria;p_Proteobacteria;c_Betaproteobacteria; o_Burkholderiales
mag.67f	*Nitrosomonas sp.*	96.21	1.07	2699433	86	49348	49.6	2525	GCA_044507425.1	k_Bacteria;p_Proteobacteria;c_Betaproteobacteria; o_Nitrosomonadales;f_Nitrosomonadaceae;g_Nitrosomonas
mag.96f	*Microbacterium sp.*	95.96	1.77	3281568	64	105589	70.8	3184	GCA_044507445.1	k_Bacteria;p_Actinobacteria;c_Actinobacteria; o_Actinomycetales;f_Microbacteriaceae;g_Microbacterium
mag.105f	*Nitrospiraceae bacterium*	95.85	2.73	4574078	7	1060767	57.9	4308	GCA_044507505.1	k_Bacteria;p_Nitrospirae;c_Nitrospira; o_Nitrospirales;f_Nitrospiraceae
mag.75f	*Chloroflexota bacterium*	95.65	1.5	3299792	643	6126	62	3409	GCA_044507415.1	k_Bacteria;p_Chloroflexi
mag.102f	*bacterium*	95.6	4.4	3354201	337	15175	66.3	3133	GCA_044507285.1	k_Bacteria
mag.3f	*Rubrivivax sp.*	95.18	4.01	3915138	226	28928	69.9	3815	GCA_044507405.1	k_Bacteria;p_Proteobacteria;c_Betaproteobacteria; o_Burkholderiales;f_Rubrivivax
mag.27f	*Actinomycetales bacterium*	95.09	1.16	2064323	33	152184	63.8	2001	GCA_044507265.1	k_Bacteria;p_Actinobacteria;c_Actinobacteria; o_Actinomycetales
mag.45f	*Acidobacteriota bacterium*	94.58	6.2	4588854	309	32424	70	4119	GCA_044507255.1	k_Bacteria;p_Acidobacteria
mag.65f	*Chloroflexota bacterium*	94.34	6.02	6354403	599	16665	66.8	6129	GCA_044507205.1	k_Bacteria;p_Chloroflexi
mag.77f	*Actinomycetota bacterium*	94.17	1.72	2497557	318	11252	72.8	2692	GCA_044507145.1	k_Bacteria;p_Actinobacteria;c_Actinobacteria
mag.112f	*Nitrosomonas sp.*	94.1	0.48	2134873	109	29643	49.7	1917	GCA_044507105.1	k_Bacteria;p_Proteobacteria;c_Betaproteobacteria; o_Nitrosomonadales;f_Nitrosomonadaceae;g_Nitrosomonas
mag.79f	*Acidobacteriota bacterium*	94.02	2.56	3687346	158	50014	68.5	3318	GCA_044507065.1	k_Bacteria;p_Acidobacteria
mag.108f	*Flammeovirgaceae bacterium*	93.82	0.69	3696566	341	14529	51.1	3366	GCA_044507115.1	k_Bacteria;p_Bacteroidetes;c_Cytophagia; o_Cytophagales;f_Flammeovirgaceae
mag.101f	*Myxococcales bacterium*	93.67	5.16	6386207	322	41029	67.5	5511	GCA_044507075.1	k_Bacteria;p_Proteobacteria;c_Deltaproteobacteria; o_Myxococcales
mag.52f	*Hyphomicrobiales bacterium*	93.62	6.51	3864611	369	16540	66.1	3967	GCA_044506985.1	k_Bacteria;p_Proteobacteria;c_Alphaproteobacteria; o_Rhizobiales
mag.22f	*Sphingomonadales bacterium*	93.33	2.14	2935939	92	63714	67.2	2793	GCA_044506885.1	k_Bacteria;p_Proteobacteria;c_Alphaproteobacteria; o_Sphingomonadales
mag.114f	*bacterium*	93.06	0.93	2290206	2	2250407	55.2	2255	GCA_044506935.1	k_Bacteria
mag.42f	*Sporichthya sp.*	93.05	0	5190006	120	76253	71.6	4958	GCA_044506825.1	k_Bacteria;p_Actinobacteria;c_Actinobacteria; o_Actinomycetales;f_Streptomycetaceae
mag.76f	*Mycolicibacterium sp.*	92.2	2.52	6900300	361	30933	67.3	6871	GCA_044506875.1	k_Bacteria;p_Actinobacteria;c_Actinobacteria; o_Actinomycetales;f_Mycobacteriaceae;g_Mycobacterium
mag.35f	*Propionibacteriaceae bacterium*	91.97	0.69	3275476	52	114537	69.9	3094	GCA_044506835.1	k_Bacteria;p_Actinobacteria;c_Actinobacteria; o_Actinomycetales;f_Propionibacteriaceae
mag.4f	*Mycolicibacterium sp.*	91.63	3.4	8974944	604	25332	65.6	9215	GCA_044506715.1	k_Bacteria;p_Actinobacteria;c_Actinobacteria; o_Actinomycetales;f_Mycobacteriaceae;g_Mycobacterium
mag.86f	*Comamonadaceae bacterium*	91.16	4.78	4669437	481	14145	71.2	4796	GCA_044506685.1	k_Bacteria;p_Proteobacteria;c_Betaproteobacteria; o_Burkholderiales;f_Comamonadaceae
mag.29f	*Chloroflexota bacterium*	90.37	1.83	3483106	33	169881	72.2	3263	GCA_044506615.1	k_Bacteria;p_Chloroflexi
mag.2f	*Hyphomicrobiales bacterium*	90.14	0.55	2857661	170	27757	64.8	2960	GCA_044506585.1	k_Bacteria;p_Proteobacteria;c_Alphaproteobacteria; o_Rhizobiales
mag.15f	*Hyphomicrobiales bacterium*	89.69	1.01	4183389	235	33445	68.7	4097	GCA_044506555.1	k_Bacteria;p_Proteobacteria;c_Alphaproteobacteria; o_Rhizobiales
mag.83f	*Deinococcaceae bacterium*	89.62	5.51	3037638	153	40847	71.8	2866	GCA_044506545.1	k_Bacteria;p_Deinococcus-Thermus;c_Deinococci; o_Deinococcales;f_Deinococcaceae
mag.14f	*Sphingomonadales bacterium*	89.59	1.54	3108227	177	34868	68.9	3116	GCA_044506565.1	k_Bacteria;p_Proteobacteria;c_Alphaproteobacteria; o_Sphingomonadales
mag.31f	*Clavibacter sp.*	89.11	1.68	2276334	238	14752	69.7	2396	GCA_044506455.1	k_Bacteria;p_Actinobacteria;c_Actinobacteria; o_Actinomycetales;f_Microbacteriaceae;g_Clavibacter
mag.74f	*Lysobacterales bacterium*	87.32	3.62	4568213	203	49372	61.6	4075	GCA_044506405.1	k_Bacteria;p_Proteobacteria;c_Gammaproteobacteria; o_Xanthomonadales
mag.115f	*Hyphomicrobiales bacterium*	83.43	1.59	3365867	221	21178	64	3391	GCA_044506365.1	k_Bacteria;p_Proteobacteria;c_Alphaproteobacteria; o_Rhizobiales
mag.95f	*Actinomycetota bacterium*	83.13	8.12	3577061	472	10101	70.5	3817	GCA_044506375.1	k_Bacteria;p_Actinobacteria;c_Actinobacteria
mag.64f	*Planctomycetaceae bacterium*	82.02	2.9	5321140	1221	5090	66.8	5139	GCA_044506435.1	k_Bacteria;p_Planctomycetes;c_Planctomycetia; o_Planctomycetales;f_Planctomycetaceae
mag.51f	*Thermomicrobia bacterium*	82	0	2854037	280	13822	62.3	2892	GCA_044506205.1	k_Bacteria;p_Chloroflexi;c_Thermomicrobia
mag.68f	*Rubrivivax sp.*	78.77	4.91	3589783	323	16828	69.9	3587	GCA_044506225.1	k_Bacteria;p_Proteobacteria;c_Betaproteobacteria; o_Burkholderiales;f_Rubrivivax
mag.44f	*Deinococcaceae bacterium*	76.48	0.42	2207965	59	58259	73.6	1979	GCA_044506245.1	k_Bacteria;p_Deinococcus-Thermus;c_Deinococci; o_Deinococcales;f_Deinococcaceae
mag.11f	*Hyphomicrobiaceae bacterium*	76.45	5.71	4525562	828	6968	63	4966	GCA_044506255.1	k_Bacteria;p_Proteobacteria;c_Alphaproteobacteria; o_Rhizobiales;f_Hyphomicrobiaceae

† Completeness.

∞ Contamination.

ˁ CDS protein-coding sequences/predicted genes.

In total, 99 high-quality MAGs were obtained, comprising 98 bacterial and 1 archaeal genome. The majority of MAGs originated from seawater samples (52), followed by cow dung (28), and landfill soil (19) (Table 2). Among the MAGs identified, *Sphingomonadales* bacterium (mag.37d, mag.20e, mag.32e), order *Flavobacteriales* (e.g., mag.1d and mag.52e), members of the genus *Mycobacterium*/*Mycolicibacterium* (mag.6d, mag.16d, mag.38d, mag.27e), and *Rubrivivax* sp. (mag.36d, mag.43e, mag.10e) were prominent. These taxa are particularly noteworthy because they have been previously associated with plastic and hydrocarbon biodegradation processes [[3], [4], [5]]. The presence of these taxa across diverse substrates such as seawater and terrestrial environments (cow dung and landfill soil) points to their broad metabolic versatility and potential utility in biotechnological applications targeting plastic waste remediation.

## 4 Experimental Design, Materials, and Methods

### 4.1 Sampling

Landfill soil samples (200 g each) were collected randomly from five different locations at a depth of 10–15 cm within the Luipaardsvlei Municipal Solid Waste Landfill Site, Mogale City, Gauteng, South Africa (26.00°S, 27.66°E). The individual samples were combined into a single composite soil sample (totaling 1000 g), placed in sterile zip-lock bags, and transported immediately on ice to the laboratory. Seawater samples were collected from the plastic-polluted shoreline of Durban, KwaZulu-Natal, South Africa (29.83°S, 31.03°E). Water was aseptically collected from three different sites using sterile 2 L polypropylene bottles, pooled into one composite sample, and transported on ice to the laboratory for subsequent analyses. Additionally, equal weights of fresh, 15-day, and 30-day composted cow dung were collected from the Dairy Unit of the Agricultural Research Council (ARC) Farm in Pretoria, Gauteng, South Africa (26.10°S, 27.02°E). The cow dung samples were homogenized into a single composite sample (500 g) and stored in sterile zip-lock bags. Upon arrival at the laboratory, all samples were stored at 4°C and utilized for microcosm enrichment experiments within three days of collection.

### 4.2 Microcosms enrichment experiments

Enrichment experiments were conducted using 500 mL Erlenmeyer flasks, each containing 200 mL of sterile mineral salts medium supplemented with 1 g of inoculum (landfill soil or cow dung), 1 mL of seawater, and 1 g of polyethylene terephthalate (PET) plastic polymers as the sole carbon and energy source. The PET used was standard granular PET beads (Pcode: 102397981) obtained from Sigma-Aldrich (catalog numbers: 429252; Sigma-Aldrich, USA). Cultures were established in triplicate and incubated for 180 days at 30°C with continuous agitation at 160 rpm, following the protocol adapted from Edwards et al. [6], with minor modifications to optimize for extended incubation under oligotrophic conditions. Flasks were loosely capped to maintain aerobic conditions, and sterile water was periodically added to compensate for evaporative loss. During the incubation, pH was monitored monthly to assess potential acidification due to microbial metabolism, and adjusted if necessary, using sterile NaOH or HCl to maintain a pH range of 7.0–7.5. No additional carbon or nutrient sources were supplied during the experiment to ensure selection pressure favored PET-degrading microbial populations.

Throughout the enrichment, strict aseptic techniques were employed to minimize contamination and ensure that observed biodegradation activities were attributable to the introduced inocula. Control flasks containing mineral salts medium and PET but no inoculum was also incubated under identical conditions to monitor abiotic degradation processes.

### 4.3 DNA extraction, library preparation, and shotgun sequencing

Following the 180-day incubation period, DNA was extracted from each enrichment consortium in triplicate, and the resulting extracts were subsequently pooled to obtain representative composite samples. Ambient (environmental) DNA was extracted from 5 mL aliquots of the enrichment cultures using the NucleoSpin® Soil DNA Extraction Kit (Macherey-Nagel, supplied by Fisher Scientific UK Ltd., Leicestershire, UK), following the manufacturer's standard protocol with no modifications. Extracted DNA samples were quantified using a Qubit 4 Fluorometer (Thermo Fisher Scientific, USA) and assessed for integrity via 1% (w/v) agarose gel electrophoresis. High-quality DNA samples were immediately stored at −79 °C until further processing.

For library construction, the MGIEasy Universal DNA Library Prep Set V1.0 (MGI Tech Co., China) was utilized, following the manufacturer’s guidelines to prepare paired-end libraries suitable for high-throughput sequencing. Library quality and insert size distributions were evaluated prior to sequencing using an Agilent 2100 Bioanalyzer (Agilent Technologies, USA). Shotgun metagenomic sequencing was performed at Agricultural Research Centre (ARC), Pretoria, South Africa, on the DNBSEQ-G400® platform (MGI Tech Co., China) generating 150 bp paired-end reads.

### 4.4 Metagenome sequence quality control, and assembly of MAGs

Raw sequencing reads generated from shotgun metagenomics were subjected to quality control using Fastqc (v0.12.0) [7] and Trimmomatic (v0.40) [8]. Adapter sequences were removed, and low-quality bases (Phred score <20) were trimmed; reads falling below the quality threshold were also discarded to ensure the retention of high-fidelity sequences.

High-quality reads were subsequently assembled de novo using metaSPAdes (v3.15.3) [9], a de Bruijn graph-based assembler optimized for metagenomic data. Assembly quality was evaluated with QUAST (v5.2) [10], which provided key metrics including N50 values, total number of contigs, and maximum contig lengths. Additionally, QUAST was used to compare assembled contigs against reference genomes to identify potential misassembles or gaps.

Binning of assembled contigs was performed using a combination of CONCOCT (v1.1) [11], MetaBAT2 [12], and MaxBin2 (v2.0) [13], based on sequence coverage patterns and tetranucleotide composition, with a minimum contig length threshold set at 2,500 bp. To improve bin quality, bins were dereplicated and integrated using the DAS Tool (v1.1.2) [14]. Bin quality was further assessed using CheckM (v1.0.18) [15], which provided estimates of completeness and contamination. High-quality MAGs were defined as those with fewer than 500 contigs, an N50 value exceeding 20,000 bp, completeness greater than 50%, and contamination less than 10% [2]. Taxonomic classification of the MAGs was performed using GTDB-Tk (v3.4.2) [16], and genome annotation was conducted with Prokka (v1.14.5) [17]. Functional annotation of the MAGs was subsequently carried out using the DRAM pipeline (v0.1.2) [18] to predict metabolic and ecological functions.

## Limitations

Not applicable.

## Ethics Statements

The authors have consulted the publisher's ethics in publishing standards and believe the manuscript meets these standards.

## CRediT authorship contribution statement

**Aubrey Dickon Chigwada:** Conceptualization, Investigation, Formal analysis, Writing – original draft, Visualization, Writing – review & editing. **Henry Joseph Oduor Ogola:** Conceptualization, Writing – review & editing, Formal analysis, Visualization. **Memory Tekere:** Conceptualization, Writing – review & editing, Supervision.

## Data Availability

NCBI SRAShotgun metagenomics analysis of microbial consotia with PS,PE, and PET biodegradation properties (Original data)
